# 4-Chloro-*N*-phenyl­benzene­sulfonamide

**DOI:** 10.1107/S1600536811015108

**Published:** 2011-04-29

**Authors:** K. Shakuntala, Sabine Foro, B. Thimme Gowda

**Affiliations:** a Department of Chemistry, Mangalore University, Mangalagangotri 574 199, Mangalore, India; b Institute of Materials Science, Darmstadt University of Technology, Petersenstrasse 23, D-64287 Darmstadt, Germany

## Abstract

In the crystal of the title compound, C_12_H_10_ClNO_2_S, the asymmetric unit contains two independent mol­ecules. The N—C bonds in the C—SO_2_—NH—C segments have *gauche* torsions with respect to the S=O bonds. The mol­ecules are twisted at the S atoms with C—SO_2_—NH—C torsion angles of −53.8 (3) and −63.4 (3)° in the two mol­ecules. The benzene rings are tilted relative to each other by 69.1 (1) and 82.6 (1)°. The dihedral angle between the sulfonyl benzene rings of the two independent mol­ecules is 23.7 (2)°. The crystal structure features inversion-related dimers linked by N—H⋯O hydrogen bonds.

## Related literature

For hydrogen-bonding preferences of sulfonamides, see: Adsmond & Grant (2001 ▶). For our study of the effect of substituents on the structures of *N*-(ar­yl)-amides, see: Gowda *et al.* (2004 ▶); on the structures of *N*-(ar­yl)aryl­sulfonamides, see: Shakuntala *et al.* (2011**a* ▶,b* ▶); and on the oxidative strengths of *N*-chloro,*N*-aryl­sulfonamides, see: Gowda & Kumar (2003 ▶).
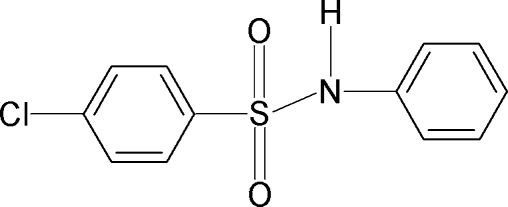


## Experimental

### 

#### Crystal data

C_12_H_10_ClNO_2_S
*M* *_r_* = 267.72
Triclinic, 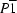

*a* = 10.206 (1) Å
*b* = 10.900 (1) Å
*c* = 13.461 (2) Å
α = 68.19 (1)°
β = 87.64 (2)°
γ = 67.08 (1)°
*V* = 1271.1 (3) Å^3^
*Z* = 4
Mo *K*α radiation
μ = 0.45 mm^−1^
*T* = 293 K
0.40 × 0.36 × 0.30 mm

#### Data collection

Oxford Diffraction Xcalibur diffractometer with a Sapphire CCD detector
Absorption correction: multi-scan (*CrysAlis RED*; Oxford Diffraction, 2009 ▶) *T*_min_ = 0.840, *T*_max_ = 0.876
8487 measured reflections
4831 independent reflections
2470 reflections with *I* > 2σ(*I*)
*R*_int_ = 0.018

#### Refinement

*R*[*F* ^2^ > 2σ(*F* ^2^)] = 0.047
*wR*(*F* ^2^) = 0.133
*S* = 0.93
4831 reflections
313 parameters
2 restraints
H atoms treated by a mixture of independent and constrained refinement
Δρ_max_ = 0.33 e Å^−3^
Δρ_min_ = −0.29 e Å^−3^

### 

Data collection: *CrysAlis CCD* (Oxford Diffraction, 2009 ▶); cell refinement: *CrysAlis RED* (Oxford Diffraction, 2009 ▶); data reduction: *CrysAlis RED*; program(s) used to solve structure: *SHELXS97* (Sheldrick, 2008 ▶); program(s) used to refine structure: *SHELXL97* (Sheldrick, 2008 ▶); molecular graphics: *PLATON* (Spek, 2009 ▶); software used to prepare material for publication: *SHELXL97*.

## Supplementary Material

Crystal structure: contains datablocks I, global. DOI: 10.1107/S1600536811015108/nc2229sup1.cif

Structure factors: contains datablocks I. DOI: 10.1107/S1600536811015108/nc2229Isup2.hkl

Supplementary material file. DOI: 10.1107/S1600536811015108/nc2229Isup3.cml

Additional supplementary materials:  crystallographic information; 3D view; checkCIF report

## Figures and Tables

**Table 1:** Hydrogen-bond geometry (Å, °)

*D*—H⋯*A*	*D*—H	H⋯*A*	*D*⋯*A*	*D*—H⋯*A*
N1—H1N⋯O1^i^	0.84 (2)	2.17 (2)	3.010 (3)	175 (3)
N2—H2N⋯O3^ii^	0.89 (2)	1.99 (2)	2.867 (4)	167 (3)

Symmetry codes: (i) 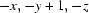; (ii) 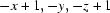.

**Fig. 1.**
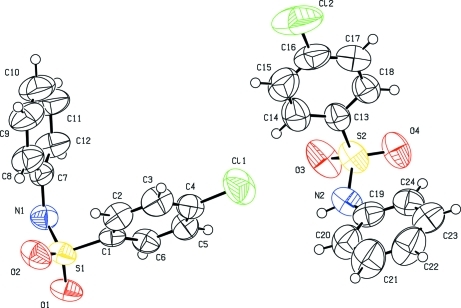
Molecular structure of (I), showing the atom labelling scheme and displacement ellipsoids are drawn at the 50% probability level.

**Fig. 2.**
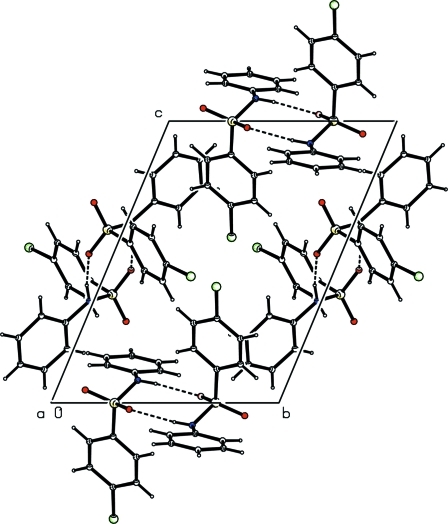
Molecular packing of (I) with hydrogen bonding shown as dashed lines.

## supplementary crystallographic information

### Comment

The sulfonamide moieties are the constituents of many biologically important compounds. The hydrogen bonding preferences of sulfonamides has been investigated (Adsmond & Grant, 2001). As a part of studying the substituent effects on the structures and other aspects of this class of compounds (Gowda, & Kumar, 2003; Gowda *et al.*, 2004; Shakuntala *et al.*, 2011**a*,b*), in the present work, the crystal structure of 4-chloro-*N*-(phenyl)-benzenesulfonamide (I) has been determined (Fig.1). The asymmetric unit of the structure contains two independent molecules. The N—C bonds in the C—SO_2_—NH—C segments have *gauche* torsions with respect to the S═O bonds. The molecules are twisted at the S atom with the C—SO_2_—NH—C torsion angles of -53.8 (3)° (molecule 1) and -63.4 (3)° (molecule 2), compared to the values of 57.6 (3)° in 4-chloro-*N*-(2-chlorophenyl)-benzenesulfonamide (II) (Shakuntala *et al.*, 2011*a*) and -58.4 (3)° in 4-chloro-*N*-(3-chlorophenyl)-benzenesulfonamide (III) (Shakuntala *et al.*, 2011*b*).

The sulfonyl and the anilino benzene rings in the two independent molecules of (I) are tilted relative to each other by 69.1 (1)° in molecule 1, and 82.6 (1)° in molecule 2, compared to the values of 84.7 (1)° in (II) and 77.1 (1)° in (III).

In the crystal structure of the title compound the molecules are linked by N—H···O(S) hydrogen bonding into dimers that are located on centers of inversion (Table 1 and Fig.2).

### Experimental

The solution of chlorobenzene (10 ml) in chloroform (40 ml) was treated dropwise with chlorosulfonic acid (25 ml) at 0 ° C. After the initial evolution of hydrogen chloride subsided, the reaction mixture was brought to room temperature and poured into crushed ice in a beaker. The chloroform layer was separated, washed with cold water and allowed to evaporate slowly. The residual 4-chlorobenzenesulfonylchloride was treated with aniline in the stoichiometric ratio and boiled for ten minutes. The reaction mixture was then cooled to room temperature and added to ice cold water (100 ml). The resultant 4-chloro-*N*-(phenyl)-benzenesulfonamide was filtered under suction and washed thoroughly with cold water. It was then recrystallized to constant melting point from dilute ethanol. The compound was characterized by recording its infrared and NMR spectra.

Prism like colorless single crystals used in X-ray diffraction studies were grown in ethanolic solution by slow evaporation at room temperature.

### Refinement

The H atoms of the NH groups were located in a difference map and later restrained to the distance N—H = 0.86 (2) Å. The other H atoms were positioned with idealized geometry with C—H = 0.93 Å and refined isotropic with *U*_iso_(H) = 1.2 *U*_eq_(C) using a riding model.

### Crystal data

**Table:**

C_12_H_10_ClNO_2_S	*Z* = 4
*M**_r_* = 267.72	*F*(000) = 552
Triclinic, *P*1	*D*_x_ = 1.399 Mg m^−^^3^
Hall symbol: -P 1	Mo *K*α radiation, λ = 0.71073 Å
*a* = 10.206 (1) Å	Cell parameters from 2291 reflections
*b* = 10.900 (1) Å	θ = 2.5–28.0°
*c* = 13.461 (2) Å	µ = 0.45 mm^−^^1^
α = 68.19 (1)°	*T* = 293 K
β = 87.64 (2)°	Prism, colourless
γ = 67.08 (1)°	0.40 × 0.36 × 0.30 mm
*V* = 1271.1 (3) Å^3^	

### Data collection

**Table:**

Oxford Diffraction Xcalibur diffractometer with a Sapphire CCD detector	4831 independent reflections
Radiation source: fine-focus sealed tube	2470 reflections with *I* > 2σ(*I*)
graphite	*R*_int_ = 0.018
Rotation method data acquisition using ω and φ scans	θ_max_ = 25.7°, θ_min_ = 2.6°
Absorption correction: multi-scan (*CrysAlis RED*; Oxford Diffraction, 2009)	*h* = −12→11
*T*_min_ = 0.840, *T*_max_ = 0.876	*k* = −13→12
8487 measured reflections	*l* = −16→16

### Refinement

**Table:**

Refinement on *F*^2^	Primary atom site location: structure-invariant direct methods
Least-squares matrix: full	Secondary atom site location: difference Fourier map
*R*[*F*^2^ > 2σ(*F*^2^)] = 0.047	Hydrogen site location: inferred from neighbouring sites
*wR*(*F*^2^) = 0.133	H atoms treated by a mixture of independent and constrained refinement
*S* = 0.93	*w* = 1/[σ^2^(*F*_o_^2^) + (0.0725*P*)^2^] where *P* = (*F*_o_^2^ + 2*F*_c_^2^)/3
4831 reflections	(Δ/σ)_max_ = 0.002
313 parameters	Δρ_max_ = 0.33 e Å^−^^3^
2 restraints	Δρ_min_ = −0.29 e Å^−^^3^

### Special details

**Table:**

Experimental. CrysAlis RED (Oxford Diffraction, 2009) Empirical absorption correction using spherical harmonics, implemented in SCALE3 ABSPACK scaling algorithm.
Geometry. All e.s.d.'s (except the e.s.d. in the dihedral angle between two l.s. planes) are estimated using the full covariance matrix. The cell e.s.d.'s are taken into account individually in the estimation of e.s.d.'s in distances, angles and torsion angles; correlations between e.s.d.'s in cell parameters are only used when they are defined by crystal symmetry. An approximate (isotropic) treatment of cell e.s.d.'s is used for estimating e.s.d.'s involving l.s. planes.
Refinement. Refinement of *F*^2^ against ALL reflections. The weighted *R*-factor *wR* and goodness of fit *S* are based on *F*^2^, conventional *R*-factors *R* are based on *F*, with *F* set to zero for negative *F*^2^. The threshold expression of *F*^2^ > σ(*F*^2^) is used only for calculating *R*-factors(gt) *etc*. and is not relevant to the choice of reflections for refinement. *R*-factors based on *F*^2^ are statistically about twice as large as those based on *F*, and *R*- factors based on ALL data will be even larger.

### Fractional atomic coordinates and isotropic or equivalent isotropic displacement parameters (Å^2^)

**Table:**

	*x*	*y*	*z*	*U*_iso_*/*U*_eq_	
Cl1	0.38715 (12)	0.51207 (13)	0.41184 (9)	0.1275 (4)	
S1	−0.02351 (7)	0.72074 (7)	0.00095 (6)	0.0670 (2)	
O1	−0.11578 (19)	0.6455 (2)	0.02316 (17)	0.0804 (6)	
O2	−0.0837 (2)	0.87352 (19)	−0.04563 (18)	0.0897 (7)	
N1	0.0811 (3)	0.6608 (2)	−0.07990 (19)	0.0692 (6)	
H1N	0.089 (3)	0.576 (2)	−0.068 (2)	0.083*	
C1	0.0854 (2)	0.6661 (2)	0.1192 (2)	0.0533 (6)	
C2	0.1321 (3)	0.7597 (3)	0.1375 (3)	0.0733 (8)	
H2	0.1012	0.8548	0.0884	0.088*	
C3	0.2241 (4)	0.7129 (4)	0.2279 (3)	0.0848 (9)	
H3	0.2559	0.7757	0.2403	0.102*	
C4	0.2683 (3)	0.5731 (4)	0.2993 (2)	0.0720 (8)	
C5	0.2225 (3)	0.4798 (3)	0.2825 (3)	0.0741 (8)	
H5	0.2528	0.3850	0.3323	0.089*	
C6	0.1322 (3)	0.5259 (3)	0.1927 (2)	0.0657 (8)	
H6	0.1017	0.4619	0.1807	0.079*	
C7	0.2053 (3)	0.6890 (3)	−0.1087 (2)	0.0664 (7)	
C8	0.2085 (4)	0.8223 (3)	−0.1344 (3)	0.0948 (10)	
H8	0.1270	0.8997	−0.1335	0.114*	
C9	0.3330 (5)	0.8399 (5)	−0.1615 (3)	0.1103 (12)	
H9	0.3356	0.9293	−0.1765	0.132*	
C10	0.4510 (5)	0.7316 (6)	−0.1669 (3)	0.1183 (14)	
H10	0.5340	0.7459	−0.1866	0.142*	
C11	0.4471 (4)	0.6022 (5)	−0.1435 (4)	0.1243 (14)	
H11	0.5282	0.5263	−0.1470	0.149*	
C12	0.3244 (4)	0.5801 (4)	−0.1142 (3)	0.0973 (11)	
H12	0.3236	0.4898	−0.0982	0.117*	
Cl2	0.97275 (15)	0.38078 (14)	0.44881 (14)	0.1891 (7)	
S2	0.70362 (9)	−0.06953 (8)	0.61492 (8)	0.0866 (3)	
O3	0.6774 (3)	−0.1070 (2)	0.5286 (2)	0.1169 (9)	
O4	0.7868 (2)	−0.1795 (2)	0.71098 (18)	0.0997 (7)	
N2	0.5442 (3)	0.0186 (3)	0.6386 (2)	0.0906 (8)	
H2N	0.477 (3)	0.060 (3)	0.583 (2)	0.109*	
C13	0.7846 (3)	0.0531 (3)	0.5660 (2)	0.0719 (8)	
C14	0.7295 (4)	0.1667 (4)	0.4690 (3)	0.1014 (11)	
H14	0.6519	0.1764	0.4283	0.122*	
C15	0.7889 (5)	0.2658 (5)	0.4321 (3)	0.1218 (15)	
H15	0.7517	0.3433	0.3662	0.146*	
C16	0.9025 (5)	0.2509 (4)	0.4917 (4)	0.1077 (13)	
C17	0.9598 (4)	0.1372 (5)	0.5872 (4)	0.1026 (11)	
H17	1.0384	0.1271	0.6268	0.123*	
C18	0.9004 (4)	0.0369 (3)	0.6248 (3)	0.0848 (9)	
H18	0.9390	−0.0415	0.6900	0.102*	
C19	0.5121 (4)	0.0836 (3)	0.7156 (3)	0.0790 (9)	
C20	0.3836 (5)	0.2019 (4)	0.6914 (3)	0.1075 (12)	
H20	0.3271	0.2407	0.6259	0.129*	
C21	0.3412 (5)	0.2617 (4)	0.7696 (5)	0.1361 (16)	
H21	0.2536	0.3397	0.7574	0.163*	
C22	0.4284 (6)	0.2053 (5)	0.8630 (4)	0.1273 (15)	
H22	0.4002	0.2455	0.9144	0.153*	
C23	0.5557 (5)	0.0913 (5)	0.8823 (3)	0.1047 (11)	
H23	0.6152	0.0554	0.9460	0.126*	
C24	0.5976 (4)	0.0284 (4)	0.8086 (3)	0.0887 (10)	
H24	0.6841	−0.0513	0.8226	0.106*	

### Atomic displacement parameters (Å^2^)

**Table:**

	*U*^11^	*U*^22^	*U*^33^	*U*^12^	*U*^13^	*U*^23^
Cl1	0.1162 (8)	0.1725 (10)	0.1093 (8)	−0.0534 (7)	−0.0125 (6)	−0.0735 (7)
S1	0.0507 (4)	0.0611 (5)	0.0912 (6)	−0.0237 (4)	0.0041 (4)	−0.0300 (4)
O1	0.0537 (11)	0.0874 (13)	0.1169 (17)	−0.0383 (10)	0.0124 (10)	−0.0469 (12)
O2	0.0689 (12)	0.0559 (12)	0.1212 (18)	−0.0118 (10)	−0.0069 (12)	−0.0220 (11)
N1	0.0686 (15)	0.0743 (15)	0.0798 (17)	−0.0378 (14)	0.0106 (13)	−0.0364 (14)
C1	0.0493 (14)	0.0480 (14)	0.0737 (19)	−0.0254 (12)	0.0187 (13)	−0.0304 (13)
C2	0.093 (2)	0.0576 (16)	0.087 (2)	−0.0403 (16)	0.0212 (19)	−0.0370 (16)
C3	0.109 (3)	0.091 (2)	0.098 (3)	−0.062 (2)	0.023 (2)	−0.061 (2)
C4	0.0646 (18)	0.095 (2)	0.074 (2)	−0.0345 (17)	0.0139 (15)	−0.0481 (18)
C5	0.079 (2)	0.0653 (18)	0.079 (2)	−0.0329 (16)	0.0075 (17)	−0.0248 (16)
C6	0.0645 (17)	0.0594 (17)	0.087 (2)	−0.0356 (15)	0.0076 (16)	−0.0315 (16)
C7	0.072 (2)	0.079 (2)	0.0570 (19)	−0.0434 (18)	0.0098 (14)	−0.0228 (15)
C8	0.102 (3)	0.083 (2)	0.107 (3)	−0.052 (2)	0.029 (2)	−0.0298 (19)
C9	0.125 (3)	0.113 (3)	0.119 (3)	−0.085 (3)	0.041 (3)	−0.037 (2)
C10	0.105 (3)	0.142 (4)	0.128 (4)	−0.079 (3)	0.045 (3)	−0.046 (3)
C11	0.096 (3)	0.128 (3)	0.154 (4)	−0.051 (3)	0.058 (3)	−0.059 (3)
C12	0.092 (3)	0.097 (3)	0.122 (3)	−0.052 (2)	0.046 (2)	−0.050 (2)
Cl2	0.1382 (10)	0.1327 (10)	0.2808 (18)	−0.0760 (9)	0.0996 (11)	−0.0487 (10)
S2	0.0843 (6)	0.0634 (5)	0.1069 (7)	−0.0150 (4)	−0.0088 (5)	−0.0407 (5)
O3	0.1039 (17)	0.1019 (16)	0.150 (2)	−0.0112 (14)	−0.0269 (16)	−0.0819 (16)
O4	0.1068 (17)	0.0676 (13)	0.1103 (18)	−0.0200 (12)	−0.0262 (14)	−0.0312 (13)
N2	0.0723 (18)	0.0866 (18)	0.113 (2)	−0.0273 (15)	−0.0021 (15)	−0.0417 (17)
C13	0.0665 (19)	0.0609 (18)	0.077 (2)	−0.0088 (15)	0.0022 (17)	−0.0320 (17)
C14	0.096 (3)	0.083 (2)	0.099 (3)	−0.017 (2)	−0.006 (2)	−0.025 (2)
C15	0.113 (3)	0.092 (3)	0.105 (3)	−0.014 (3)	0.026 (3)	−0.010 (2)
C16	0.087 (3)	0.088 (3)	0.137 (4)	−0.031 (2)	0.053 (3)	−0.040 (3)
C17	0.075 (2)	0.115 (3)	0.128 (4)	−0.041 (2)	0.023 (2)	−0.055 (3)
C18	0.077 (2)	0.076 (2)	0.090 (3)	−0.0201 (19)	0.0030 (19)	−0.0307 (18)
C19	0.083 (2)	0.066 (2)	0.101 (3)	−0.0424 (19)	0.030 (2)	−0.0350 (19)
C20	0.117 (3)	0.075 (2)	0.101 (3)	−0.022 (2)	0.012 (2)	−0.020 (2)
C21	0.147 (4)	0.083 (3)	0.128 (4)	−0.007 (3)	0.022 (4)	−0.029 (3)
C22	0.162 (4)	0.094 (3)	0.110 (4)	−0.033 (3)	0.043 (3)	−0.045 (3)
C23	0.112 (3)	0.113 (3)	0.098 (3)	−0.050 (3)	0.033 (2)	−0.046 (2)
C24	0.081 (2)	0.092 (2)	0.104 (3)	−0.044 (2)	0.015 (2)	−0.039 (2)

### Geometric parameters (Å, °)

**Table:**

Cl1—C4	1.727 (3)	Cl2—C16	1.732 (4)
S1—O2	1.4152 (19)	S2—O4	1.406 (2)
S1—O1	1.4301 (18)	S2—O3	1.432 (2)
S1—N1	1.625 (3)	S2—N2	1.625 (3)
S1—C1	1.746 (3)	S2—C13	1.750 (3)
N1—C7	1.424 (3)	N2—C19	1.422 (4)
N1—H1N	0.844 (16)	N2—H2N	0.889 (17)
C1—C6	1.375 (3)	C13—C18	1.366 (4)
C1—C2	1.380 (3)	C13—C14	1.368 (4)
C2—C3	1.371 (4)	C14—C15	1.367 (5)
C2—H2	0.9300	C14—H14	0.9300
C3—C4	1.365 (4)	C15—C16	1.359 (5)
C3—H3	0.9300	C15—H15	0.9300
C4—C5	1.362 (4)	C16—C17	1.358 (5)
C5—C6	1.358 (4)	C17—C18	1.378 (5)
C5—H5	0.9300	C17—H17	0.9300
C6—H6	0.9300	C18—H18	0.9300
C7—C12	1.351 (4)	C19—C24	1.346 (4)
C7—C8	1.376 (4)	C19—C20	1.376 (5)
C8—C9	1.373 (4)	C20—C21	1.405 (6)
C8—H8	0.9300	C20—H20	0.9300
C9—C10	1.341 (5)	C21—C22	1.357 (6)
C9—H9	0.9300	C21—H21	0.9300
C10—C11	1.343 (5)	C22—C23	1.353 (5)
C10—H10	0.9300	C22—H22	0.9300
C11—C12	1.380 (5)	C23—C24	1.371 (5)
C11—H11	0.9300	C23—H23	0.9300
C12—H12	0.9300	C24—H24	0.9300
			
O2—S1—O1	119.51 (12)	O4—S2—O3	119.25 (14)
O2—S1—N1	108.62 (13)	O4—S2—N2	110.50 (16)
O1—S1—N1	104.49 (12)	O3—S2—N2	103.86 (15)
O2—S1—C1	107.78 (12)	O4—S2—C13	107.49 (15)
O1—S1—C1	109.11 (12)	O3—S2—C13	108.16 (16)
N1—S1—C1	106.65 (12)	N2—S2—C13	106.99 (13)
C7—N1—S1	123.44 (19)	C19—N2—S2	125.8 (2)
C7—N1—H1N	116 (2)	C19—N2—H2N	115 (2)
S1—N1—H1N	109 (2)	S2—N2—H2N	114 (2)
C6—C1—C2	119.1 (3)	C18—C13—C14	120.2 (3)
C6—C1—S1	119.92 (19)	C18—C13—S2	120.4 (2)
C2—C1—S1	120.9 (2)	C14—C13—S2	119.4 (3)
C3—C2—C1	120.2 (3)	C15—C14—C13	119.7 (4)
C3—C2—H2	119.9	C15—C14—H14	120.1
C1—C2—H2	119.9	C13—C14—H14	120.1
C4—C3—C2	119.2 (3)	C16—C15—C14	119.9 (4)
C4—C3—H3	120.4	C16—C15—H15	120.0
C2—C3—H3	120.4	C14—C15—H15	120.0
C5—C4—C3	121.2 (3)	C17—C16—C15	120.9 (4)
C5—C4—Cl1	119.3 (3)	C17—C16—Cl2	118.9 (4)
C3—C4—Cl1	119.5 (2)	C15—C16—Cl2	120.2 (4)
C6—C5—C4	119.6 (3)	C16—C17—C18	119.5 (4)
C6—C5—H5	120.2	C16—C17—H17	120.3
C4—C5—H5	120.2	C18—C17—H17	120.3
C5—C6—C1	120.7 (2)	C13—C18—C17	119.8 (3)
C5—C6—H6	119.7	C13—C18—H18	120.1
C1—C6—H6	119.7	C17—C18—H18	120.1
C12—C7—C8	118.6 (3)	C24—C19—C20	122.3 (3)
C12—C7—N1	118.0 (3)	C24—C19—N2	122.2 (3)
C8—C7—N1	123.3 (3)	C20—C19—N2	115.4 (4)
C9—C8—C7	119.4 (3)	C19—C20—C21	117.4 (4)
C9—C8—H8	120.3	C19—C20—H20	121.3
C7—C8—H8	120.3	C21—C20—H20	121.3
C10—C9—C8	121.8 (4)	C22—C21—C20	119.8 (4)
C10—C9—H9	119.1	C22—C21—H21	120.1
C8—C9—H9	119.1	C20—C21—H21	120.1
C9—C10—C11	118.7 (4)	C23—C22—C21	120.9 (4)
C9—C10—H10	120.6	C23—C22—H22	119.5
C11—C10—H10	120.6	C21—C22—H22	119.5
C10—C11—C12	121.0 (4)	C22—C23—C24	120.4 (4)
C10—C11—H11	119.5	C22—C23—H23	119.8
C12—C11—H11	119.5	C24—C23—H23	119.8
C7—C12—C11	120.4 (3)	C19—C24—C23	119.2 (4)
C7—C12—H12	119.8	C19—C24—H24	120.4
C11—C12—H12	119.8	C23—C24—H24	120.4
			
O2—S1—N1—C7	62.1 (2)	O4—S2—N2—C19	53.3 (3)
O1—S1—N1—C7	−169.3 (2)	O3—S2—N2—C19	−177.7 (3)
C1—S1—N1—C7	−53.8 (2)	C13—S2—N2—C19	−63.4 (3)
O2—S1—C1—C6	165.8 (2)	O4—S2—C13—C18	−5.0 (3)
O1—S1—C1—C6	34.6 (2)	O3—S2—C13—C18	−135.0 (2)
N1—S1—C1—C6	−77.7 (2)	N2—S2—C13—C18	113.7 (3)
O2—S1—C1—C2	−17.9 (3)	O4—S2—C13—C14	176.0 (3)
O1—S1—C1—C2	−149.1 (2)	O3—S2—C13—C14	46.0 (3)
N1—S1—C1—C2	98.6 (2)	N2—S2—C13—C14	−65.3 (3)
C6—C1—C2—C3	0.0 (4)	C18—C13—C14—C15	−1.4 (5)
S1—C1—C2—C3	−176.3 (2)	S2—C13—C14—C15	177.6 (3)
C1—C2—C3—C4	−0.1 (5)	C13—C14—C15—C16	0.1 (6)
C2—C3—C4—C5	−0.2 (5)	C14—C15—C16—C17	1.1 (6)
C2—C3—C4—Cl1	178.6 (2)	C14—C15—C16—Cl2	−176.7 (3)
C3—C4—C5—C6	0.6 (5)	C15—C16—C17—C18	−1.1 (6)
Cl1—C4—C5—C6	−178.2 (2)	Cl2—C16—C17—C18	176.8 (3)
C4—C5—C6—C1	−0.7 (4)	C14—C13—C18—C17	1.4 (5)
C2—C1—C6—C5	0.4 (4)	S2—C13—C18—C17	−177.6 (2)
S1—C1—C6—C5	176.8 (2)	C16—C17—C18—C13	−0.2 (5)
S1—N1—C7—C12	139.2 (3)	S2—N2—C19—C24	−32.0 (4)
S1—N1—C7—C8	−43.1 (4)	S2—N2—C19—C20	151.6 (3)
C12—C7—C8—C9	−2.1 (5)	C24—C19—C20—C21	−2.1 (5)
N1—C7—C8—C9	−179.9 (3)	N2—C19—C20—C21	174.3 (3)
C7—C8—C9—C10	2.2 (6)	C19—C20—C21—C22	2.1 (6)
C8—C9—C10—C11	−1.1 (7)	C20—C21—C22—C23	−0.4 (7)
C9—C10—C11—C12	0.0 (7)	C21—C22—C23—C24	−1.5 (6)
C8—C7—C12—C11	1.0 (5)	C20—C19—C24—C23	0.3 (5)
N1—C7—C12—C11	178.9 (3)	N2—C19—C24—C23	−175.9 (3)
C10—C11—C12—C7	0.0 (6)	C22—C23—C24—C19	1.6 (5)

### Hydrogen-bond geometry (Å, °)

**Table:**

*D*—H···*A*	*D*—H	H···*A*	*D*···*A*	*D*—H···*A*
N1—H1N···O1^i^	0.84 (2)	2.17 (2)	3.010 (3)	175 (3)
N2—H2N···O3^ii^	0.89 (2)	1.99 (2)	2.867 (4)	167 (3)

Symmetry codes: (i) −*x*, −*y*+1, −*z*; (ii) −*x*+1, −*y*, −*z*+1.
